# Genetics and genomic medicine in Ecuador

**DOI:** 10.1002/mgg3.192

**Published:** 2015-12-21

**Authors:** César Paz‐y‐Miño, María J. Guillen Sacoto, Paola E. Leone

**Affiliations:** ^1^ Instituto de Investigaciones BiomédicasUniversidad de las AméricasQuitoEcuador; ^2^ National Human Genome Research InstituteNational Institutes of HealthBethesdaMarylandUSA

## Abstract

Genetics and genomic medicine in Ecuador.

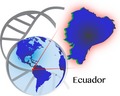

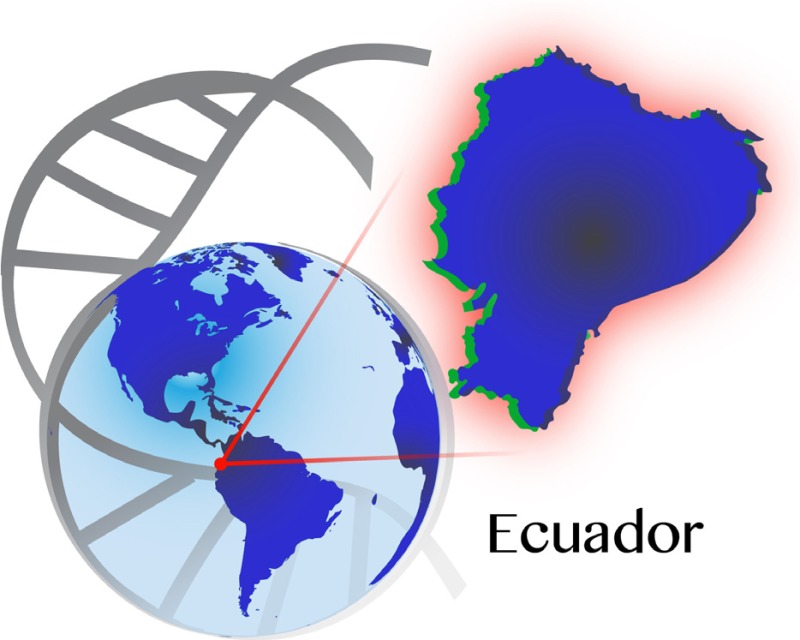

## Introduction

**Figure 1:**
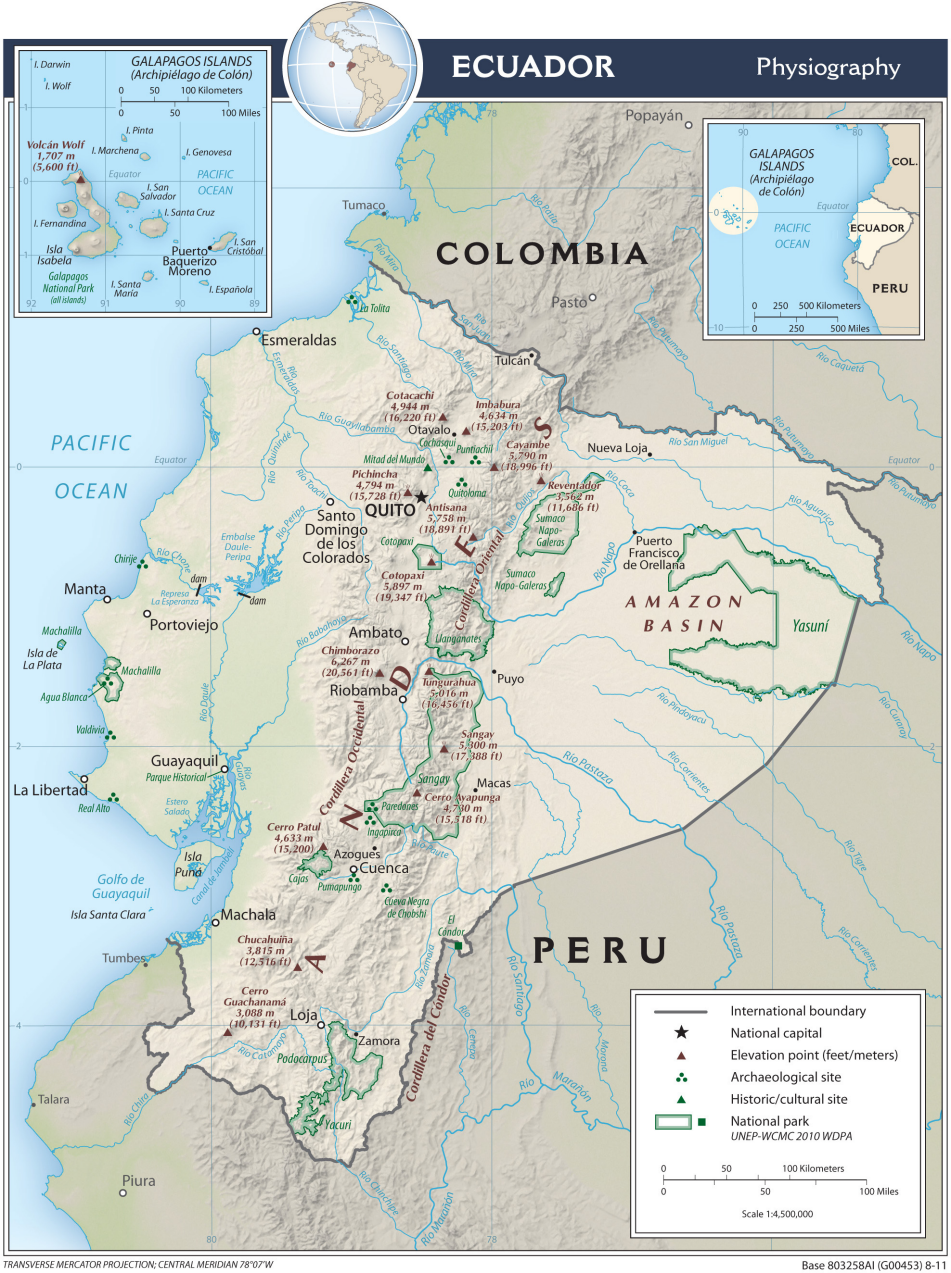
Political map of Ecuador.

Ecuador is located between latitudes 2°N and 5°S and limits the Pacific Ocean on the west, Colombia on the north, and Peru on the south and east (Fig. 1). Its capital, Quito, stands at 2850 m (9350 ft.) above sea level. The country has four different geographic regions, the Coast (west of the Andes), the Sierra (Interandean highlands), the Amazon (east of the Andes), and the Insular region (Galapagos Islands). It is also divided into 24 provinces; six in the Coast, 11 in the Sierra, six in the Amazon, and one in the Insular region.

Ecuador's surface area is 256,369 km^2^ (98,985 sq miles). The population in the last National Census in 2010 was 14,483,499 and it is estimated to be above 15 million as of 2015 (National Institute of Census and Statistics, INEC, www.inec.gob.ec). Life expectancy in Ecuador is 75.6 years (CIA, World Fact Book). Its gross domestic product (GDP) per capita was $5932 with a GDP growth rate of 4.05% in 2013 (Ecuadorian Ministry of Finances).

## Population Diversity

In 2010, 71.9% of Ecuadorians self‐identify as Mestizos, 6.1% as white, 7% as Amerindians, and 7.2% as African descendants. Mestizos live in all urban areas of the country, whereas Amerindians and Afro‐Ecuadorians tend to occupy more limited rural areas.

The Ecuadorian population originates from a complex mixture of a large number of Amerindian tribes, Africans and Europeans (Baeta et al. 2013). It is marked by historic migratory and colonization events that created population bottlenecks and from a genetic standpoint have influenced genetic flow and genetic drift (Raff et al. 2011; Paz‐y‐Miño and Burgos Figueroa 2015). The first Americans appear to have come from Asia and spread through the entire continent about 15,000 years ago (Tamm et al. 2007; Yang et al. 2010; Reich et al. 2012). These people settled in different areas adapting to their surroundings and developing their own culture in situ, giving rise to the Amerindian race. Analysis of Y chromosome DNA has also shown some Asian haplotypes that are present in South American Amerindians but not in native populations of Central and North America, which supports the idea of a second migration, this time by sea through the Pacific Ocean, about 6000 years ago (Roewer et al. 2013). Analysis of mitochondrial DNA show that modern Amerindians have less haplogroups when compared to ancient settlers, which confirms the dramatic effect of early dispersion following the initial migration (Raff et al. 2011).

The next large historic migration came with the Spanish conquest, which gave rise to Mestizos, a hybrid population with European, African, and Amerindian influences. The specific genetic makeup of Ecuadorian Mestizos has been studied using STR (short tandem repeat), SNPs (single‐nucleotide polymorphisms), Y chromosome markers, and mitochondrial DNA. Analysis of Y‐chromosome DNA in Mestizos show the Q1a3a haplogroup, shared by all South American Amerindians, and three other haplogroups, R1b, E1b1b, and TE, commonly found in European and African men, respectively (Gaviria et al. 2013). Mitochondrial DNA studies begin with the analysis of the Cytochrome b gene in Mestizos, Native American, Cholos, and African‐Americans, relating common polymorphisms to the possible origin of each population (Paz‐y‐Miño et al. 2008a). It is clear that the mitochondrial contribution came primarily from Amerindian women, with varying frequencies of specific haplotypes depending on the population (Baeta et al. 2012). The case is not that easy when discussing markers involving the nuclear genome. STR analysis of autosomic markers showed 73% were of Amerindian origin, 19% European, and 8% African (González‐Andrade et al. 2007); whereas Y chromosome markers showed over 70% of European, 22% Amerindian, and 2% African contribution in Mestizos (González‐Andrade et al. 2007; Gaviria et al. 2013). Studies looking at the X‐chromosome have also shown significant differences (Baeta et al. 2013; Gaviria et al. 2013). This large differences are in part explained by the population studied, suggesting that there is a larger than expected diversity, and pointing to additional contribution from Asia, Oceania, China, and Northern Europe (Paz‐y‐Miño et al. 2007a). It also highlights the need to use appropriate databases when studying Ecuadorian populations. To our knowledge, the most complete database that includes the 22 loci recommended by the ISFG (International Society of Forensic Genetics) was published by Gaviria et al. 2013 and studied 1800 Ecuadorian Mestizos.

Among the Amerindians, there are at least 32 distinct tribes, Kichwa being the most populous and found throughout the Sierra (INEC, www.inec.gob.ec). A remarkable population is the Waoroni, a small tribe deep in the Amazon region, who did not come in contact with western civilization until the 1950s. As a result, this small group of people, 2416 individuals as of 2010, has a unique genetic makeup, distinct from other Amerindians in the country and America in general. Studies of mitochondrial DNA revealed only two haplotypes, A2 (Cardoso et al. 2008; Baeta et al. 2009) and D1 (Baeta et al. 2009), with 94% of the Waorani population sharing the A2 haplotype (Baeta et al. 2009; Cardoso et al. 2012). The same was observed when studying Y‐chromosome DNA. Waorani men share six haplotypes that are not found in any of 499 populations studied around the world at that time (González‐Andrade et al. 2009a). This showed very little genetic variability, as expected for a small group of people, geographically isolated, and with frequent consanguineous marriages (Baeta et al. 2009; Cardoso et al. 2012).

The Afro‐Ecuadorian population is also quite interesting in the sense that it is related to the Guineans, but it is closer to the Kichwas and Spaniards than to the Africans (Paz‐y‐Miño 2012a).

## Healthcare in Ecuador

The public health sector has the largest coverage in the country. The National Health System includes all public health centers in addition to supporting private centers that have been qualified and registered and that serve the population at no charge to the patient since 2008. However, waiting times can be long and some centers lack resources to attend the high demand for services. Private hospitals and clinics are available in every specialty and subspecialty, although they are expensive. In 2014, there were 17 physicians per 10,000 people, with higher numbers in the Sierra and urban areas in general.

Health insurance coverage in 2014 was 41.4% (INEC, www.inec.gob.ec). This includes private insurance as well as social security through different programs of the Ecuadorian Institute of Social Security (IESS). For individuals with formal employment, enrollment in the social security system is mandatory. Self‐employed or unemployed people can also voluntarily enroll in the system and have access to health services through the IESS.

In the period between 2000 and 2011, the top five causes of death in Ecuador were hypertension, stroke, diabetes, cirrhosis, and other liver diseases, and violence (INEC, www.inec.gob.ec). According to the last population Census, in 2010, 5.64% of Ecuadorians had a disability. 24% of those were identified as having intellectual disability, 62% moderate or severe (Ministry of Education Information System, SIME, sime.educacion.gob.ec). In 2009, the Vice‐presidency of Ecuador lead the “Manuela Espejo Solidarity Mission” aimed, among other things, to identify the causes of intellectual disabilities in the country. In its summary report published in November 2011, 8% of patients with intellectual disability have a diagnosis of cerebral palsy. Forty‐two percent are due to chromosomal abnormalities, 16% are monogenic, and 43% are multifactorial (Manuela Espejo Solidarity Mission 2013).

## Genetics in Ecuador

**Figure 2:**
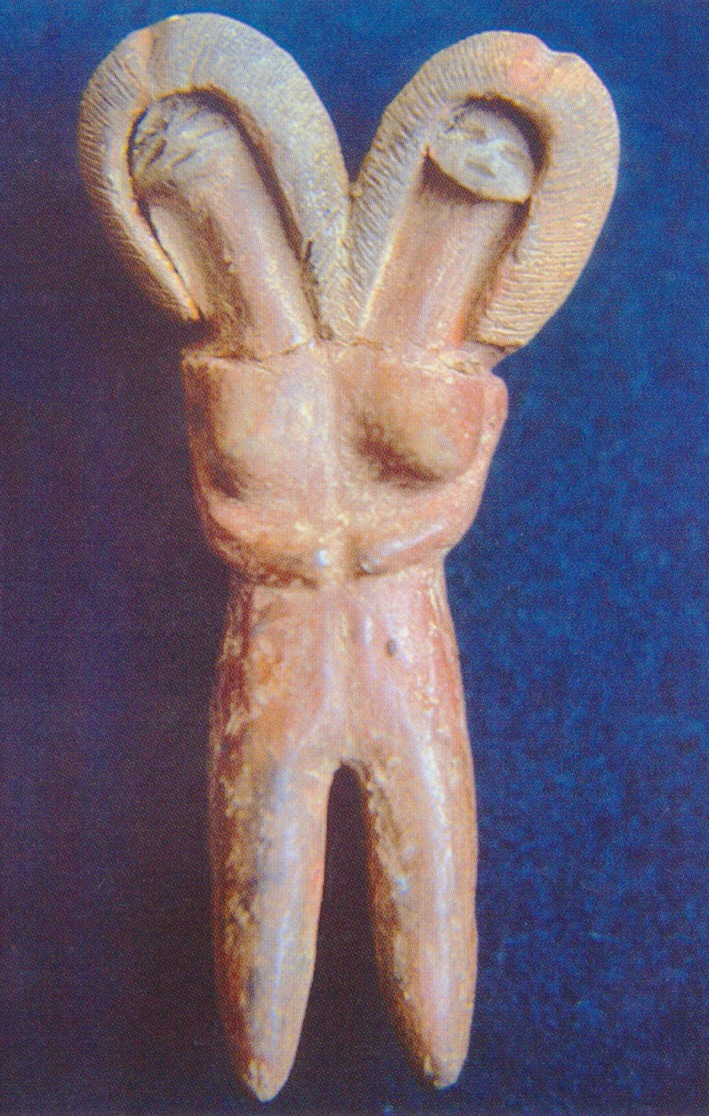
Culture Valdivia figurine showing bicephalus, a women with two heads. From: Hermida Bustos, Enrique. Temas de Paleopatología Ecuatoriana. Academia Nacional de Historia, Quito 2013.

**Figure 3:**
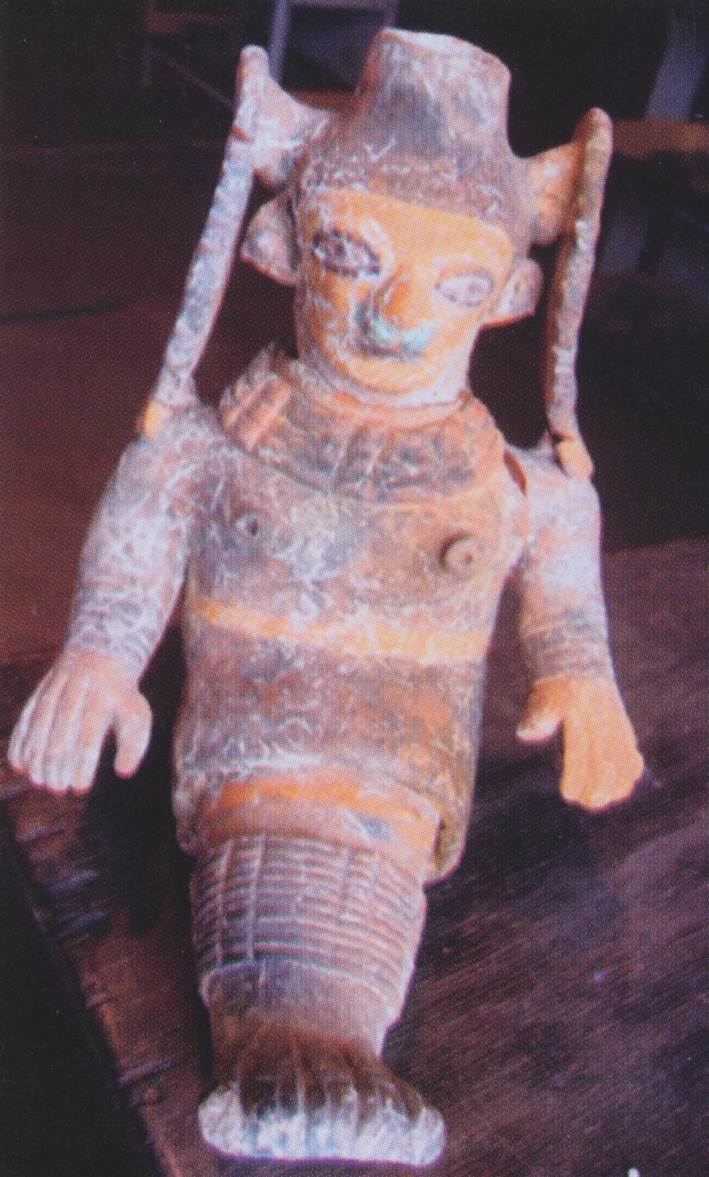
Sirenomelia. From: Hermida Bustos, Enrique. Temas de Paleopatología Ecuatoriana. Academia Nacional de Historia, Quito 2013.

Inhabitants of what today is Ecuador have documented the presence of genetic disease for thousands of years. The Valdivians (3500–1800 BC) made pottery representations of conjoined twins (Fig. 2), the Chorrera (1300‐300 BC) of sirenomelia (Fig. 3), the Machalillas (1500 BC) of polydactyly and ectrodactyly, and the Tolitas (500 BC) of giants and dwarfs. Charles Darwin developed his theory on the evolution of species based on observations made in the Galapagos Islands in 1835; which ignited the work of several Ecuadorian scientist like Father Solano, with his Biological Species in Ecuador in 1839; Adolfo Hidalgo N., Pathologic Inheritance in 1915; and Guillermo Torres, Anatomic Abnormalities in 1921. The first Ecuadorian book in Genetics, Inheritance and Men, was published by Robert Hoffstetter in 1947 (Paz‐y‐Miño and López‐Cortés 2014a; Leone and Paz‐y‐Miño 2015). However, a study in suburban communities and shamans of Ecuador showed that both groups are poorly informed about genetics (Paz‐y‐Miño et al. 2006).

Human Genetics, as a branch of medicine, truly begins in Ecuador at the Armed Forces hospital in 1983. Genetics services were later opened in Quito first, and then in Guayaquil and Cuenca; however, Ecuador is one of the few countries in Latin America where the Government and the Public Health System do not count with genetic services in their hospitals (Paz‐y‐Miño 2012a). In 1989, the Ecuadorian Society of Human Genetics was formed and in 2007 the first Ecuadorian Congress in Human Genetics took place. At its beginnings, the services offered were focused on clinical diagnosis, cytogenetics, prenatal diagnosis, and genetic counseling. Today, we have added to those services molecular cytogenetics, metabolic and biochemical genetics, molecular genetics, ontogenetics, filiation, and forensics (Paz‐y‐Miño 1998, 2004a).

The World Health Organization recommends the presence of a geneticist for each 100,000 inhabitants in Latin‐America, which means Ecuador needs 15, we currently have 10. Three centers have a formal genetics department, and two more had a clinical geneticist on staff and performed inpatient and outpatient consults. Five centers have their own genetics laboratory with capability to perform karyotypes, FISH (fluorescence in situ hybridization), HPLC (high performance liquid chromatography), and target mutation analysis by PCR (polymerase chain reaction), SSCP (single‐strand conformation polymorphisms), RFLP (restriction fragment length polymorphism), Sanger sequencing, and arrays analysis. The subject of genetics is taught in most medical schools, starting at the Universidad Central del Ecuador in 1988 (Leone and Paz‐y‐Miño 2015).

Private practice begins in late 1990s with the study of a large number of diseases but with a small number of tests performed (Leone and Paz‐y‐Miño 2015).

## Genetic Disorders in Ecuador

As mentioned earlier, almost half of the cases of intellectual disability in Ecuador are due to a chromosomal abnormality (Manuela Espejo Solidarity Mission). This is the result of a combination of health policies that do not allow prenatal diagnosis and therapeutic termination of pregnancies (Penchaszadeh 2002), and gene–environment interactions that influence the development of such conditions in the first place, ethnic background, geographic location, and toxic exposures (Paz‐y‐Miño 2012a). In a cohort of 2636 cases analyzed by G‐banding karyotyping between 1998 and 2012, the most common abnormality was trisomy 21 (Paz‐y‐Miño et al. 2012). A striking particularity is the incidence of mosaic trisomy 21, 10% in the registry, compared to the expected 2–4% (Jorde et al. 2006). Maternal age and ethnic background probably have an impact on these numbers, but there is also some evidence suggesting that hypoxia and high altitude play a role (Paz‐y‐Miño 2012a).

Among congenital malformations, cleft lip and palate is the most common, microtia is six times more common than in any other Latin American country, and the incidence of hip dysplasia is also four times higher (Montalvo et al. 2006). Some of these observations again can be explained, at least in part, by the high altitude of most of the inhabited areas in the Ecuadorian Sierra (Castilla et al. 1999; González‐Andrade et al. 2010).

The incidence of specific mutations has also been studied in Ecuador and helps highlight the differences in our population with respect of the rest of Latin America and the world. In the case of cystic fibrosis, F508del, the most common mutation in all populations including Hispanics in the United States (Watson et al. 2004), is found in only 25–37% of patients studied. The authors also looked at the seven most common variants in Caucasians after F508del and did not find any in our population (Paz‐y‐Miño et al. 1999). The three common variants in the HFE gene associated with hereditary hemochromatosis type 1 were also assessed in a random population sample of Mestizos and in 12 patients with hemochromatosis. The C282Y variant was not found in any of the 224 alleles studied, whereas the frequency of H63D and S65C were significantly higher in the hemochromatosis group, similar to what is observed in Asian populations (Leone et al. 2005). We found a recurrent mutation in a cohort of patients with autosomal recessive sensorineural hearing loss, Q7X, representing 18.3% of alleles tested and associated with severe hearing loss when homozygous or compound heterozygous with another mutation (Paz‐y‐Miño et al. 2014b). This variant had previously been described only once in the literature, in an Ecuadorian child (Pandya et al. 2003). The distribution of the ApoE alleles associated with Alzheimer disease is also peculiar in the Ecuadorian population. The ApoE *ε*4 allele was present in 17.9% of affected and 10.3% of unaffected individuals versus 44% and 17% reported in Latin American and Caucasian populations, whereas the *ε*2*ε*2 genotype is absent, as expected in Native American and mixed race populations (Paz‐y‐Miño et al. 2010d). The presence and size of CAG repeats in the HTT gene responsible for Huntington Disease was analyzed and found to be in accordance with publications from the rest of the world, including the United States (Pavón‐Realpe et al. 2014).

A big push in genetics comes from SOLCA (Sociedad de Lucha Contra el Cancer), a nonprofit organization dedicated to prevention, diagnosis, and treatment of malignancies. The first molecular diagnostic tests performed in Ecuador focused on the characterization of hematologic malignancies (Leone et al. 2002; Paz‐ y Miño et al. 2002d; Paz‐y‐Miño et al. 2002c, 2003a,b, 2007c, 2010c, 2013; Zucca et al. 2013). Several studies have also shown that the incidence of tumors, both benign and malignant, is also influenced by high altitude (Rodriguez‐Cuevas et al. 1986; Vogelstein and Kinzler 1998; Paz‐y‐Miño 2002a). As such, we have conducted studies to determine the contribution of genetic factors to specific cancers in Ecuador. We have described mutations in NF2 (Paz‐y‐Miño and Leone 2000), and RB1 (Leone et al. 2003b); as well as single‐nucleotide polymorphisms that increase the risk or alter the course of the disease in meningiomas (Leone et al. 2003a), prostate cancer (Paz‐y‐Miño et al. 2009; López‐Cortés et al. 2013), lung cancer (Paz‐y‐Miño et al. 2010a), and bladder cancer (Paz‐y‐Miño et al. 2010b). We have also found polymorphisms in cancer repair genes like MSH2 and BCL2 that seem to correlate with the higher frequency of malignancies in populations exposed to toxics in Ecuador (Paz‐y‐Miño et al. 2002b).

Mutagenesis and the effects of toxics on human beings was an area of concern since the early 1990s. Paz‐y‐Miño studied the effects of radiation, clastogenic substance, pesticides, and petroleum hydrocarbons (Paz‐y‐Miño et al. 1995, 1997, 2001, 2002b; Paz‐y‐Miño et al. 2002e; Paz‐y‐Miño et al. 2004b, 2007b, 2008b, 2011; Paz‐y‐Miño et al. 2014c); Cantos and López studied heavy metals on the Ecuadorian population (Leone and Paz‐y‐Miño 2015). Also, there are several laboratories that specialize in tropical diseases (Paz‐y‐Miño 2004a), HIV (Paz‐y‐Miño et al. 2005), HPV (Paz‐y‐Miño et al. 1992; González‐Andrade and Sánchez 2009b), and Helicobacter pylori (Cabrera‐Andrade et al. 2014).

The incidence of autosomal recessive conditions is influenced by the frequency in consanguineous marriages, 1.25% overall and up to 6% in families with intellectual disability, and usually involve first cousin matings (Liascovich et al. 2001; Ministry of Education Information System, SIME, sime.educacion.gob.ec). One of such diseases is Laron syndrome or growth hormone insensitivity syndrome. Laron syndrome is characterized by extreme short stature with variable intellectual disability (MIM #262500). It is caused by biallelic mutations in the growth hormone receptor gene and is especially common in a small isolate in the province of Loja (Rosenbloom et al. 1990; Guevara‐Aguirre et al. 1991). Although affected individuals of Jewish and Ecuadorian descent share the same common mutation, p.E180; the cohort from Loja has a skewed prevalence in women and are reported to be of normal and superior intelligence (Guevara‐Aguirre et al. 1991). It has also been postulated that these individuals have longer lives, with decreased incidence of cancer and diabetes (Guevara‐Aguirre et al. 2011).

## Reproductive Law

Abortion is illegal in Ecuador, except under two circumstances: to save the life or preserve the health of the mother, or if the pregnancy is the result of rape to a woman with intellectual disability. As of August 2014, women who actively induce an abortion, or that authorize someone else to do it, could face jail time of 6 months up to 2 years. The physician who performs the abortion can be jailed for up to 7 years (Código Penal del Ecuador, Art. 150). There are no exceptions for severe malformations or incurable genetic conditions affecting the fetus.

As such, prenatal diagnosis is limited to malformations detectable by ultrasound. Invasive testing is a rare occurrence and most of Obstetricians have little experience, which in turn increases the risks for both the fetus and the mother.

## Newborn Screening Program

Newborn screening is available for four disorders: phenylketonuria, galactosemia, congenital adrenal hyperplasia and congenital hypothyroidism. The program started in December 2011 and in the first 3 years, 401,776 newborns were screened. One hundred and sixty‐one children had a positive result: 33 had congenital adrenal hyperplasia, 111 hypothyroidism, seven galactosemia, and 10 phenylketonuria (Ministry of Public Health, www.salud.gob.ec).

## Final Remarks

Ecuador faces big challenges when taking care of patients with genetic conditions. The demand for modern technologies represents a large expense for a country with a growing economy. Access to techniques such as massive parallel sequencing, arrays, FISH, among others is limited and in many cases requires sending the samples abroad.

The study of the genetic makeup of the Ecuadorian population encourages planning of our own genetic health interventions and maybe 1 day the institution of personalized medicine.

Another important challenge is a change in our laws to allow the prevention of genetic diseases, prenatal and preimplantation diagnosis and cell‐free DNA in maternal serum. In the absence of therapeutic abortions for fetal conditions, preventative actions are not concrete.

Many of the demands for attention of patients with genetic conditions, including genetic and reproductive counseling, would be solved by the National Genetics Center, which remains to be established.

## Conflict of Interest

None reported.
